# When the Algorithm Speaks First: Trust, Reliance and the Reconstruction of Clinical Metacognition

**DOI:** 10.1111/jep.70652

**Published:** 2026-10-06

**Authors:** Joel Hanhart, Anat Zohar

**Affiliations:** ^1^ Department of Ophthalmology Shaare Zedek Medical Center Jerusalem Israel; ^2^ Faculty of Medicine Hebrew University of Jerusalem Jerusalem Israel; ^3^ Seymour Fox School of Education Hebrew University of Jerusalem Jerusalem Israel

**Keywords:** artificial intelligence, clinical reasoning, diagnostic judgment, epistemic trust, metacognition, person‐centred care

## Abstract

**Aims of the Journal Addressed:**

This paper addresses three of the aims of the Journal: clinical decision making, health philosophy and person‐centred healthcare. Retinal imaging serves as the analytic case. The question at issue, what epistemic attitude a clinician may properly hold towards an algorithmic output issued for an individual patient, arises in every discipline and every health system in which such systems are deployed, and the argument is tested throughout against systems that read no images.

**Rationale, Aims and Objectives:**

Diagnostic artificial intelligence increasingly delivers a conclusion before the clinician has formed one, and medical education has responded by adding modules on the critical appraisal of algorithmic output. Such curricula rest upon two unsound foundations: they extend a form of metacognitive instruction shown not to improve diagnostic accuracy and they ask clinicians to cultivate an attitude whose defining feature is the relaxation of validation. This paper establishes what epistemic attitude a clinician may hold towards an algorithmic output and derives the form that clinical metacognition must take in consequence.

**Methods:**

Conceptual analysis, drawing upon the philosophy of trust and epistemic dependence, the cognitive science of clinical reasoning and empirical studies of artificial intelligence in clinical practice. Retinal medicine serves as the analytic case, chosen because it is the domain in which the position under contest is strongest; the argument is tested throughout against systems that process no images.

**Results:**

Trust and reliance are distinct attitudes. On the account adopted here, to trust a medical algorithm is to rely upon it while relaxing the monitoring of the conditions that make it reliable; trust is reliance from which validation has been withdrawn. Regulatory clearance, institutional adoption and published performance supply legitimate grounds for trusting the assemblage that produces a system, yet none of them, singly or together, warrants reliance upon its output for an individual patient. Three asymmetries separate algorithmic from human epistemic dependence: the algorithm can be neither interrogated nor held to account; its errors fall on whole classes of patients at once; and its warrant holds of a population while the decision is owed to a person. The metacognition this situation demands is knowledge, not disposition, and stands untouched by the evidence against generic metacognitive instruction.

**Conclusions:**

Clinical metacognition requires reconstruction around three competences: calibrated reliance, awareness of distributed epistemic labour, and vigilance towards confidence‐masked uncertainty. Each is content‐dependent, teachable and assessable. Since none can be exercised where institutions supply neither the means of interrogation nor the time it takes, the reform of education and the reform of clinical governance are, in this respect, a single undertaking.

## 1 Introduction

In a growing share of clinical encounters the algorithm has already spoken before the physician has begun. No judgment is formed and then checked against a machine; a conclusion has been produced in advance of the encounter, by a system whose reasoning cannot be inspected, about a patient the clinician has yet to examine. It arrives unbidden, in every encounter.

Medicine has met this development in the register of literacy. Training programmes are acquiring modules on the critical appraisal of algorithmic output, the reading of performance metrics and the recognition of what machine learning cannot do. Reasonable as it is, the response falls short on two independent counts.

It rests, to begin with, on no settled concept. No validated framework governs the competencies that deploying diagnostic artificial intelligence in specialty training requires; the proposals so far advanced share no account of the competence they mean to instil; and accreditation standards carry no matching content [1, 2]. In a national survey of ophthalmologists and residents, more than three quarters endorsed structured education in the subject while fewer than one in six judged themselves prepared for it [3]. Curricula are being assembled under institutional pressure, ahead of the idea that would make them coherent. It rests, further, on an unsound foundation, since the generic metacognitive instruction these modules extend has been shown across a decade of evidence to leave diagnostic accuracy untouched [4]. A storey has been added to a structure already straining under its own weight.

The concept that is missing, we argue, is not the one the field has assumed. The prevailing proposal is to teach clinicians calibrated trust in algorithmic systems. Trust and reliance, however, are distinct attitudes, and trust, in this setting, is best understood as reliance from which validation has been withdrawn. Once that is seen, the clinician's difficulty takes on a definite shape. Regulatory clearance, institutional adoption and published validation supply abundant and legitimate grounds for trusting the apparatus that produced a system. They supply no grounds, by themselves, for relying on what it reports of the patient before us. The new situation multiplies the reasons for trust and withdraws the means of validation, leaving no boundary between the two.

The remedy is reconstruction, where the field has attempted addition. Clinical metacognition has to be rebuilt around a body of knowledge, content‐dependent and teachable, in place of a disposition to reflect. And because the conditions under which that knowledge can be exercised are supplied by institutions rather than curricula, the reconstruction is a task for clinical governance as much as for education.

We develop the argument through retinal imaging, the domain that tests it most severely: the imaging substrate is standardised, the validation datasets are large, the prospective real‐world evidence is among the most extensive in medicine, and the regulatory pathway is settled. In April 2018 the United States Food and Drug Administration granted De Novo authorisation to IDx‐DR, the first autonomous diagnostic system in any medical specialty to read fundus photographs (images of the retina, at the back of the eye) without physician review at the point of care [5]. Here, if anywhere, algorithmic diagnosis could pass for a sharper instrument that leaves clinical reasoning otherwise intact, and additive reform might suffice. The argument therefore proceeds a fortiori: what holds under the most favourable conditions holds with greater force where conditions are worse. To show that it does not depend on the image, we test it at each stage against systems that read none. The argument is conceptual throughout and reports no new data. The empirical studies it draws upon were identified in the course of a scoping review of artificial intelligence in medical retina training conducted by one of us [2], and extended by targeted searching in adjacent specialties for work on clinician‐algorithm interaction. We have retained those whose designs bear on the question at issue and have stated the limits of each where they weigh on the inference. No systematic coverage is claimed, nor could it be: on a literature growing at its present rate none would hold for long. The studies are here to give a conceptual framework purchase on practice, and to expose it to correction.

### 1.1 Three Systems, Three Epistemic Positions

Precision is a condition of the argument, for the term artificial intelligence gathers under one word systems that stand in very different relations to clinical judgment.

The systems in use occupy different epistemic positions. Autonomous ones return a diagnostic conclusion with no physician review, as IDx‐DR and EyeArt do when they issue a referral decision from a fundus photograph in primary care. Assistive ones return an intermediate output that the clinician then reads: automated segmentation (the outlining of anatomical layers) on optical coherence tomography, the cross‐sectional scan of the retina; detection of macular fluid or of geographic atrophy; algorithmic grading of retinopathy severity [6]. Generative ones produce prose, and are entering clinical documentation, where they may omit or fabricate clinically material detail [7].

The diagnostic systems, autonomous and assistive alike, are trained by supervised learning on labelled images, and this bears on the argument twice. The labels are themselves expert judgments, carrying the disagreements and uncertainties of the experts who supplied them, so that the ground truth against which such systems are validated is itself contested knowledge, as Lebovitz and colleagues have shown [8]. The models, moreover, are fixed at the point of clearance. They do not learn from the clinician who overrules them. To dissent from an autonomous system is to correct a single decision and improve nothing beyond it; the objection leaves no mark on the system, or on the institution, unless a channel has been built to receive it.

The figure most often quoted is the area under the receiver operating characteristic curve (AUROC), a measure of how well a system separates two classes across a test population. The metrics are themselves being refined; AUROC serves here as the measure longest established in the clinical literature. Current deep‐learning systems for diabetic retinopathy screening reach pooled AUROC values in prospective real‐world settings ranging from 0.959 for referable disease to 0.974 for vision‐threatening disease [9]. The inferences such a figure permits, and those it forbids, are the burden of this paper.

### 1.2 Where Generic Instruction Stops

Clinical metacognition has long been understood as the capacity to monitor, regulate and reflect upon one's own reasoning [4, 10]. The understanding is sound; its application in medical education has arguably gone astray.

Within metacognition, Flavell distinguished two components [11]. Metacognitive knowledge is a person's knowledge of their own cognition: its conditions, its vulnerabilities, the circumstances in which it holds and those in which it gives way. Metacognitive skill is the running capacity to monitor and regulate cognition as it unfolds. Metacognitive knowledge is tied to content; metacognitive skill travels more freely across domains; and the clinical literature has largely left the consequences of that difference unexplored [12]. Reflection, in Schön's sense and in the sense clinical education took from him, is one component of metacognitive skill; it does not exhaust metacognition, though the two are often run together [13]. On this footing the dual‐process tradition built a coherent account of diagnostic expertise, one that cast metacognition as the detection of moments when fast automatic reasoning should be interrupted and checked by slower deliberation. It thereby gave educators a rationale for reflective exercises and debiasing [14].

A decade of evidence has undone that rationale. Norman, Pelaccia, Wyer and Sherbino have argued, marshalling that evidence, that generic metacognitive instruction, whether it teaches cognitive biases, critical‐thinking frameworks or general reflective practice, yields no consistent gain in diagnostic accuracy [4]. Their account is that the two processes of dual‐process theory are epiphenomenal: effects of the kind of knowledge being retrieved, not faculties operating upon it [4, 15]. To teach a clinician to consider alternatives, without supplying the structured knowledge of which alternatives exist and when each is plausible, yields no reliable improvement. One intervention in this family does show reproducible benefit: deliberate reflection, the structured examination of which features of a case support the leading hypothesis and which count against it [16]. It works because it mobilises specific, case‐relevant knowledge.

The finding sits within a wider account of expertise. Dreyfus and Dreyfus placed higher‐stage expertise in embodied pattern recognition that resists explicit rules, a point Polanyi had put in general form: the expert knows more than can be told [17, 18]. The chess studies traced the grandmaster's power to knowledge laid down over tens of thousands of hours, not to general strategic skill [19, 20]. A clinician at proficiency runs no checklist; recognition is, before anything else, an act of perception. Generic instruction is on two counts ill‐suited to this: it assumes an inward transparency that mature expertise does not possess, and it treats thinking as a faculty that improves apart from the knowledge it works upon.

The moral is plain, and easily missed. A general capacity to monitor and check can be taught with some freedom from content; knowledge of the conditions under which one's own recognition is likeliest to betray one cannot, being wedded to content through and through. A clinician taught to slow down and check has gained the first. A clinician who knows where their own pattern recognition tends to fail has gained the second. Generic instruction cannot furnish it and algorithmic diagnosis now exacts it in a register no curriculum yet reaches.

Clinical reasoning has always turned on a question the clinician puts to themselves: am I reasoning correctly about this patient? Algorithmic diagnosis adds one they were never trained to ask, and that no amount of clinical experience answers: is this output reliable for this patient?

The two are not versions of one another. Appraising one's own pattern recognition draws on self‐knowledge built through practice, the experiential base, the remembered errors, the feel for a familiar case. Appraising an algorithmic output draws on knowledge of an entirely different provenance: how the system was trained, where it is known to fail, and how this patient stands to the population it learned from [2]. The first is a harvest of clinical experience; the second cannot be gathered that way at all.

Current curricula have treated the newer question as a more taxing form of the older one. That conflation is the founding error. The rest of this paper sets out what should stand in its place.

### 1.3 Opacity Without Accountability

Medicine has always been epistemically distributed. The radiologist, the pathologist and the laboratory scientist each place an authority between the treating clinician and the diagnostic conclusion, and each does so through reasoning the clinician cannot fully inspect. Hardwig showed that such dependence is the ordinary condition of knowledge rather than a defect in it [21]. The expert who recognises a lesion at a glance cannot state the grounds of the recognition, and the colleague who accepts the report trusts a process they cannot reconstruct. Opacity is where professional work has always lived, and an objection presses at once: if the algorithm is one more opaque authority among many, why should it call for anything new?

Bearman and Ajjawi give the objection its sharpest form. They define an algorithmic interaction as one in which a computational artefact supplies a judgment that cannot be traced, observe that human expertise is untraceable in much the same way, and conclude that the effort to see inside the black box is misconceived; learners need the capacity to work with opacity itself [22, 23]. Their premise is sound: human expertise is indeed opaque. Their conclusion holds for a great deal of professional work and breaks down for clinical diagnosis, where the opacity of a colleague and the opacity of a deployed model part company along three lines.

One line is interrogation and accountability. The radiologist who cannot say why a nodule looks malignant can still be asked, argued with, and held responsible. Hardwig's dependence rests on the expert's character and on their membership of a community that sanctions incompetence. Lebovitz and colleagues observed what happens when the source has neither. In an ethnographic study of radiologists using algorithmic tools across three departments of a teaching hospital, algorithmic outputs regularly diverged from the clinician's own reading, and in the face of that divergence no common ground with the system existed or could be built [24]. Where clinicians resolved it, they did so through what Lebovitz calls interrogation practices: effortful work by which the expert relates their own claim to the algorithm's. Those practices appeared in one of the three departments. In the other two, clinicians accepted the output without scrutiny or dismissed it without engagement, a pattern she names unengaged augmentation and finds indistinguishable in effect from automation. The determinant was the evidentiary substrate: chest computed tomography let radiologists scroll adjacent slices, alter contrast and compare with prior imaging, and these affordances gave purchase on an unexplained mark, whereas a single hand radiograph gave almost none, and interrogation collapsed. Autonomous retinal screening is the limiting case. When IDx‐DR returns a referral decision on a fundus photograph, the clinician holds a verdict, an image, and no means of checking the one against the other.

Another is the shape of error. A radiologist's misses are idiosyncratic, born of this clinician's fatigue and this clinician's blind spots, and largely uncorrelated from one patient to the next. A model's misses are systematic. They fall on the subgroups the training distribution underrepresented, and they fall on every member of that subgroup, in every institution running the same model. Obermeyer and colleagues demonstrated this for a risk algorithm governing the care of millions [25]. Wong and colleagues found the same for the Epic Sepsis Model, deployed across hundreds of American hospitals, which reached an AUROC of 0.63 in external validation and missed 67 percent of septic patients [26]. Neither system reads an image. The structure of the failure is the same.

The last is the form of the warrant. A pooled AUROC of 0.96 in prospective settings entails nothing about whether this patient, with this fundus and this demographic profile, has been correctly classified. Clinical judgment is owed to an individual; the algorithm's credential was earned across a population. The old regime placed an anchor between the two in grading standards framed in clinical concepts and applicable to single patients. That anchor has been removed and nothing has replaced it.

One further feature governs how often all of this arises. A colleague is consulted when doubt runs high, which is regularly but not always. The algorithm volunteers an opinion on every case, solicited or not, and delivers it before the clinician can ask.

The distributed cognition of medicine is not new; its normative anchoring has given way. Such an authority cannot be interrogated, cannot be held to account, errs by whole classes of patients at once, and answers to a population where the patient needs an answer of their own. Table 1 sets the two regimes side by side; taken singly, several of these features admit of incremental accommodation, but taken together they constitute the reconfiguration this paper describes.

**Table 1:** Diagnostic judgment before and after algorithmic diagnosis: An epistemic reconfiguration.

	Pre‐algorithmic regime	Algorithmic regime
Source of the contributed judgment	A human agent who has examined this patient, knows the clinical question, and may be asked why	A statistical function trained on a historical population, unaware of the question posed, offering no account of its output
Grounds of dependence	The contributor's epistemic character and their membership of a community that sanctions incompetence	Published performance figures, regulatory clearance and institutional procurement, none of which attaches to the individual case
Form of the warrant	Grading standards formulated in clinical concepts and applicable to an individual patient	Discriminability across a test population, from which no individual‐case inference follows
Shape of error	Idiosyncratic to the clinician; largely uncorrelated across patients	Systematic; falling upon whole subgroups, in every institution running the same model
Locus of accountability	The contributor, who bears professional and legal responsibility for the contribution	The clinician, who retains responsibility for a conclusion substantially shaped by a contribution they cannot inspect
Occasion of contribution	Solicited when the clinician's doubt is high	Delivered on every case, before the clinician has asked
Effect on competence	Competence developed through practice remains available	Conditions for the formation of independent competence progressively withdrawn

*Note:* Feature by feature, the table opposes the pre‐algorithmic regime to the algorithmic one. No single row is decisive; their convergence is.

Such an authority no longer prompts the question whether its verdict is right. It prompts a prior one: what attitude may the clinician properly hold towards it?

### 1.4 The Proper Objects of Trust and Reliance

The word *trust* moves through this field with great freedom. Regulators require trustworthy artificial intelligence, editorials urge clinicians to trust algorithms appropriately, curricula offer to teach calibrated trust in machine output. The word carries commitments. Drawing those out recasts the educational problem.

Ordinary usage already marks a difference that the field has let slip. We rely on a bridge; we trust a colleague. When a bridge gives way it has failed. When a colleague gives way they have betrayed us, for betrayal is possible only where a commitment was undertaken and could have been kept. Trust, then, is owed to a party who holds commitments and can be answerable for breaking them; reliance asks only for a regularity that holds. An algorithm holds no commitments and answers for nothing. This is why Hatherley concludes that such systems can be relied upon and can be reliable, yet can be neither trusted nor trustworthy [27]. On that view, to teach clinicians calibrated trust in an algorithm is to teach a confusion.

The objection can be answered without being dismissed. Grant that the artefact holds no commitments; a workable notion of trust survives the concession. To trust a medical algorithm, in the account Ferrario and colleagues give, is to rely on it while relaxing the monitoring and control of the things that make it reliable [28]. Trust so understood is a thinner species of reliance, where intuition expects a warmer one: reliance with the checking relaxed.

Perhaps those who commend calibrated trust intend by it what we have called calibrated reliance, in which case the quarrel is one of words. Words of this sort do not lie idle. Trust, on the account before us, is reliance with the checking loosened, so a curriculum undertaking to calibrate a clinician's trust in an output has fixed upon the one attitude whose mark is the withdrawal of the validation that curriculum exists to secure. The remedy asks for no retreat, only for the two words to be given back their work: reliance holds the question of warrant open in each case, and trust is owed to those who can be called upon to answer.

The reformulation earns its place by what it explains. Automation bias is usually described as a lapse of attention under pressure; here it acquires a precise shape, as the moment monitored reliance tips into trust and the clinician lets go of the controls that reliance depended on. The tipping is easily triggered, and an explanation alone will do it. When a visual explanation accompanied correct algorithmic advice, physicians outside their specialty grew more accurate and rated the advice more highly, while specialists on the same task gained little; the explanation moved the non‐expert further than the expert [29]. Accuracy improved, and the study records no cost; that an explanation may serve as licence to relax validation as readily as aid to performing it is a hypothesis the finding accommodates and does not establish. A randomised study bears on it directly. Clinicians shown a systematically biased model lost 11.3 percentage points against their own baseline, and image‐based explanations, whose focus on clinically irrelevant features was evident on inspection, recovered none of that loss; two thirds of participants were unaware that a model could be biased in this way [30]. Predictions from a standard model, in the same study, improved accuracy. The explanations assisted validation where little validation was called for and failed where it was. A further trial found physicians given a large language model reasoning no better than without it, though the model alone outperformed them all; prompting familiarity and time were constrained by the design, so the result speaks to the handling of the outputs and not to their quality [31].

Where, then, does trust belong? In the sociotechnical assemblage within which the artefact operates: the developer who chose the training population, the regulator who licensed the device, the validation regime that produced the figures, the institution that procured it, and the local monitoring that tracks, or fails to track, its behaviour in this population. These parties hold commitments, can default on them, and can be called to account. They are proper objects of trust. That trust may be well or badly placed. Towards the output for the patient in front of them, what the clinician extends is reliance, warranted to the degree that it is validated.

The competence we mean to describe follows from holding the two apart. Warranted trust in the wider apparatus licenses no reliance on its output for the patient at hand. The unnoticed passage from the one to the other is the characteristic error of algorithmic diagnosis. The situation conspires to produce that passage, for the marks of an apparatus worth trusting, its validation across populations, its regulatory clearance, its adoption by the institution, are the same marks that settle nothing about this fundus and this patient. A further circumstance leaves the passage unopposed. A high confidence score presents itself as the system's testimony to its own certainty, and the one thing the clinician most needs to be told, that the system has left the ground on which it was validated, is the one thing that score cannot convey.

Hatherley's stronger claim, that such systems can never be trusted at all, remains open, and nothing below turns on rejecting it. We take the weaker one because it keeps what is sound in the clinician's sense that some expectations of these systems are legitimate, and lodges those expectations where they can be honoured: in the apparatus, which can answer for itself, never in the output, which cannot.

### 1.5 Towards a New Metacognition

The metacognition we propose to put in place answers three epistemic conditions that current frameworks were not built to meet.

#### 1.5.1 Calibrated Reliance

The capacity to judge what reliance an algorithmic output warrants for this patient, in this context, and to keep that judgment apart from any trust the surrounding apparatus may deserve.

Consider a resident reading the fundus of a 58‐year‐old with type 2 diabetes of 15 years' standing, screened in primary care with no history of retinopathy. IDx‐DR returns a negative screen. Nothing here departs from the conditions the system was authorised under. The pivotal trial reported 87.2 percent sensitivity and 90.7 per cent specificity, reaching its case numbers by enriching for disease, so that the mean glycated haemoglobin of the trial population stood well above that of the participants enrolled without enrichment; the prevalence against which those figures were obtained is not the prevalence of the clinic in which the resident sits, and the predictive value of a negative screen moves with it [5]. The image is within the acceptance criteria and near the lower edge of the quality range on which the system was validated. Every ground for trusting the assemblage is present: the device is cleared, the institution has procured it, the trial is published. None of these grounds tells the resident whether to rely on this output.

The judgment it calls for holds several kinds of knowledge in a single grip: of retinal disease, of how this particular system was trained and where it is known to fail, and of how this patient stands to the population the model learned from. Current curricula supply the clinical part and gesture in general terms at the algorithmic one, leaving their union, which is where calibration actually occurs, for the clinician to improvise.

The gap will narrow. Trial populations broaden, enrichment designs give way to validation in the settings of use, and the distance between the resident's patient and the studied population diminishes with each generation of evidence. It is a real improvement, and it leaves the problem in place. Performance metrics, however well optimised, are population‐statistical, while the decision they inform is owed to an individual. The mismatch survives any improvement in the data, and it is this mismatch that a reconfigured metacognition must address. The same holds for the other improvements now in view. Better explanations, post‐deployment monitoring, tighter probabilistic calibration and models that adapt after release each strengthen the grounds for trusting the apparatus, and none converts that trust into a warrant for the individual output. The distinction between the two remains operative under each.

The instruments, meanwhile, earn their place. In a randomised screening trial of more than a hundred thousand women, algorithmic support for mammographic reading raised sensitivity from 73.8 to 80.5 percent at unchanged specificity and without excess interval cancers [32]. Standard models improved clinician accuracy in the vignette study noted above [30], and dermatologists offered a decision aid took its advice more readily when the advice was sound than when it was mistaken [33]. Each of these findings stands, and the distinction stands with them. A gain in sensitivity across a screened population reports what an instrument does to a rate; the clinician signing a conclusion has to know what it has done to this fundus, and no aggregate will say. A system performing at that level is the one whose individual outputs are least often examined; improvement in aggregate performance therefore makes the judgment we describe more exacting.

#### 1.5.2 Awareness of Distributed Epistemic Labour

The capacity to reconstruct, within a given encounter, how the epistemic work behind the conclusion was divided between human and algorithmic agents.

A single retinal encounter may pass through several of the systems described above, each contributing at a different point and none of them inspectable. The clinician who signs the conclusion takes responsibility for a process they did not wholly perform. Van der Niet and Bleakley warned that technologies of this kind reconfigure epistemic jurisdiction, altering who is authorised to know what, and under what conditions [34]. A clinician who cannot say which part of a diagnosis they produced cannot discharge the supervisory responsibility that law and professional ethics assign them. That responsibility is not the physician's alone. Nurses, radiographers and technicians work under the same systems, with narrower epistemic jurisdiction and less recourse when an output gives them pause. Signing for a conclusion one cannot interrogate presses hardest where the standing to question it is slightest. Our vocabulary follows physician training because the empirical work we draw upon studies physicians almost throughout; the analysis reaches further.

The stakes exceed accountability. Stiegler's account of proletarianisation describes how technical systems absorb the operations that once constituted a practice, hollowing out the situated understanding that gave them meaning [35]. A finding from endoscopy is consistent with it. In an observational multicentre study, endoscopists working under continuous exposure to algorithm‐assisted colonoscopy recorded an adenoma detection rate on their unassisted procedures of 22.4 percent, against 28.4 percent before that exposure began [36]. The design leaves room for confounding by secular trend, case mix and observation, and detection rate reports detection, not attention. A prospective trial that assessed performance once the system had been withdrawn found no such decrement, its authors noting that the two designs differ in whether exposure is concurrent with the measurement [37]. Taken together they locate the effect where our argument places it, in the scrutiny a clinician sustains while an algorithm reads alongside them, and leave open how far it outlasts the instrument.

Teaching this competence means cultivating the habit of asking, in any algorithmically mediated encounter, which part of the conclusion the clinician produced, which part the system proposed, and whether the clinician is in a position to vouch for the system's part. The habit must be built deliberately, for it extends no existing clinical reasoning practice.

#### 1.5.3 Vigilance Towards Confidence‐Masked Uncertainty

At issue here is a form of uncertainty that clinical training does not prepare physicians to meet, because it wears the face of its opposite. The competence it calls for is knowledge before it is temperament: the clinician must know what a confidence score reports and what it cannot, so that a steady number is read as information about the model, never as reassurance about the patient.

Clinicians are trained on uncertainty that is felt. The ambiguous fundus lesion produces hesitation, and hesitation triggers the slowing‐down that Moulton and colleagues identified as a marker of expert judgment [38]. Zepf and colleagues find the same mechanism in trainees, who enter metacognitive reflection through discomfort and disorganisation [39]. The signal is affective, and it works.

A confidence score is not that signal. As noted above, the score indexes rank within the learned distribution and not the fit between the model's representation and the case at hand, so a model can return a probability of 0.94 while being systematically miscalibrated for a subpopulation, an imaging device, or an image‐quality range outside its training. The consequence is that the signal on which metacognitive engagement depends is itself suppressed. Lebovitz's radiologists bear on this obliquely. They engaged an algorithmic output when it diverged from their own reading, the divergence forcing the question; where output and reading converged, the provocation was absent, and with it the occasion to scrutinise [24]. Her analysis leaves an inference undrawn. Convergence between a clinician and a black box can manufacture subjective certainty with no matching epistemic gain, since the source of the agreement stays as opaque as in divergence, and it does so silently, in the cases nobody flags.

Vigilance of this kind is far from reflexive doubt. It asks the clinician to treat the system's agreement as no less in need of examination than its dissent, and to replace the comfortable question of how the system performs in aggregate with the harder one of whether, given its validation conditions and this patient's features, its output may be relied upon here and now.

Table 2 sets out the three competences. Its final column responds to the objection that metacognitive proposals resist assessment. Each behaviour is observable in a clinical encounter or case discussion; none reduces to an injunction to reflect.

**Table 2:** Three competences of clinical metacognition under algorithmic diagnosis.

	Epistemic operation	Unaddressed by current curricula	Assessable behaviour
Calibrated reliance	Integrating domain knowledge, meta‐algorithmic knowledge of the deployed system, and case‐specific knowledge, to determine what reliance this output warrants for this patient; holding that determination distinct from trust in the assemblage that produced the system	Trust in the regulatory, institutional and validation apparatus is treated as licensing reliance on individual outputs. The transfer is never examined, and no framework distinguishes the two attitudes	States the population a deployed system was validated on and whether the patient before them belongs to it; names a condition under which the output would not be relied upon
Awareness of distributed epistemic labour	Reconstructing, within an encounter, which contributions were made by human agents and which by algorithmic systems, and determining whether the clinician can vouch for each	Curricula address the algorithm as a tool met at a single point, and not as a series of contributions distributed across a pathway that the clinician signs	Maps the algorithmic contributions embedded in a given diagnostic pathway; identifies the point at which independent judgment was last exercised
Vigilance towards confidence‐masked uncertainty	Interrogating high‐confidence outputs, and interrogating agreement on the same terms as disagreement; distinguishing a system's expressed confidence from its reliability for the case at hand	Instruction targets algorithmic error and disagreement. Convergence, which produces subjective certainty without epistemic gain, is treated as a resolution and not as an event requiring scrutiny	Interrogates a case in which the algorithm confirmed their own judgment; articulates what the confirmation did and did not establish

*Note:* Each competence is a distinct epistemic operation, unmet by current training and observable in practice.

### 1.6 Knowledge, and Therefore Teachable

The proposal answers the decade of evidence that has told against metacognitive instruction [4]. It does so by being a proposal about knowledge.

That evidence condemns metacognition cut loose from content: monitoring taught as a portable procedure, on the assumption that thinking sharpens independently of the matter it works upon. A metacognition anchored in content escapes that verdict: knowledge of the conditions under which one's own recognition holds and gives way, and of what a given instrument can and cannot establish for the patient at hand. Content of this order admits of specification, instruction and examination.

The three competences we propose are knowledge of just this sort. Meta‐algorithmic knowledge is a body of content the trainee learns, as they learn pharmacology: the population a system was trained on, the imaging conditions under which it falters, its recorded failure modes, the standing of this patient to the distribution the model learned from. To grasp that trust belongs to the apparatus and reliance to the output is to know something about one's own epistemic position. Both are, in kind, continuous with the one intervention the evidence already vindicates.

A second and more sympathetic body of work must also be engaged, for it has lately given medical education its most searching account of reflective practice [40, 41]. On that account, metacognitive reflection is enacted through inquiry, adaptive action, and the reading of underlying patterns. The account is a considerable achievement, and it was worked out without a thought to algorithmic mediation. It rests on three conditions that the older clinic supplied so reliably that no one thought to state them. Inquiry, which bids the reasoner walk a colleague through their reasoning, assumes a reasoning that can be walked through. Adaptive action, roused by the discomfort of a gap in one's knowledge, assumes that the gap is felt, and trainees do indeed enter reflection through just such disorientation and unease [39]. The reading of patterns assumes that a pattern is still there to be read.

Algorithmic diagnosis unsettles all three at once. Its output cannot be walked through. Its confidence score smothers the discomfort that would have called reflection into play. And its pre‐reading has discerned the pattern already, surrendering the conclusion before the clinician's own act of discernment can occur [35]. The finest available theory of reflective clinical practice was built for a clinic that algorithmic diagnosis is quietly taking apart.

An observation from the empirical literature meets this from the other side: clinicians who cannot examine the reasoning behind an algorithmic decision are shut out of the reflective cycle altogether [13, 24]. That observation invites a further step. The shared exploration that reflective practice places at its centre cannot happen between clinician and machine, since the two, as those radiologists found, hold no common ground and can build none. It must move elsewhere, out of that barren pairing and into the company of other clinicians, where inquiry again has a partner.

Metacognition emerges from the sceptical case intact in exactly the form algorithmic diagnosis now requires, and its reconciliation with the reflective tradition, by way of Flavell's distinction, is itself a step in the argument rather than a preliminary to it. Knowledge, unlike disposition, can be examined. A trainee can be asked which population a deployed system was validated on, and whether the patient before them belongs to it. An injunction to reflect leaves nothing to ask.

### 1.7 Teaching and Governance as One

Each of these competences depends on conditions that only the clinical and institutional setting can supply. No module added to the curriculum will supply them.

To interrogate a system the clinician needs something to interrogate it with. The radiologists discussed above examined algorithmic outputs where the evidentiary substrate gave them purchase; where it did not, they gave up [24]. A screening system that answers a fundus photograph with a bare referral offers no such purchase. Calibrated reliance is then impossible in principle. The first obligation here precedes teaching and falls in the procurement office: a hospital acquiring a diagnostic system should ask what recourse it leaves the clinician who doubts a given verdict, treating a system that leaves none as a system that turns its users into signatories.

It also requires time, which is the first thing a busy service withdraws. Interrogation collapsed in that study where the evidentiary substrate gave clinicians nothing to work with [24]. A second condition operates alongside it, one the ethnography was not designed to measure. Examining an unexplained output takes minutes, and a service that has no minutes will not find them. To train epistemic supervisors for services that grudge the minutes supervision takes is to prepare them for work they will not be permitted to do. The reform of education and the reform of the institution are, in this respect, a single task.

Since inquiry with the machine is impossible, disagreements between clinician and algorithm should be gathered up as material for collective teaching, instead of being filed away as discrepancies to be reconciled. Where expert judgment and machine part ways, two conclusions stand, neither guaranteed; to adjudicate between them the clinician must put into words the reasoning that ordinarily stays beneath words. Such moments are among the richest that training affords, forcing into the open the pattern recognition on which expertise silently rests [16]. Their agreements deserve the same scrutiny, and are the harder case to institute, for agreement never announces itself as a problem.

Competence must be built before it can be supervised. A clinician who has never read a fundus unaided holds no standard against which an algorithmic reading might be weighed. In a saturated training environment the danger is subtler than the wearing‐away of an existing skill: the skill never forms at all. This failure has a name, never‐skilling: the failure of a foundational competence to develop in a trainee formed under algorithmic assistance, as distinct from the erosion of one already held [42, 43]. That it may fail to form is, by its nature, harder to demonstrate, and the window in which it could have formed will have shut before the demonstration arrives. A prospective trial bears on the distinction. Inexperienced endoscopists detected more while a detection system was running, and once it was withdrawn their unassisted performance did not differ significantly from what it had been before exposure began [37]. The trial was not designed to measure the formation of competence, and its final phase was modest in size; the pattern it records is nonetheless the one at issue, of a benefit that holds while the instrument is in place and leaves no deposit behind it.

Protected stretches of unaided diagnosis are, on these grounds, an epistemic necessity, and they cost the patient nothing. The instrument may stay in the case; the requirement is one of order. Let the trainee reach a reading and record it, and let the output follow. A supervisor arbitrates the disagreements, and the patient is managed on the two readings together. The encounter has gained a human eye and lost nothing at all. Order of this kind is the condition of calibrated reliance, for a clinician who holds no estimate of their own has nothing to weigh the system's against.

Some settings will not admit it. Where minutes decide the outcome, or where a screening system is deployed to work without an ophthalmologist at hand, the sequence has no room in which to run. Elsewhere it does, and the price is a few minutes of the trainee's reading, charged to the service rather than to the patient. The obligation falls once more upon the institution.

Order of presentation has been put to the test. In a randomised comparison, clinicians did better than with conventional resources whether the system offered its assessment before their own or after it, and overall accuracy was much the same either way [44]. The trial ran on written vignettes with a language model rather than on images, and its authors offer the results as hypothesis‐generating; they show that the order which preserves formation performs as well as its reverse.

Meta‐algorithmic knowledge, finally, belongs in the curriculum as taught content: the provenance of the training data, the populations behind the published figures, the imaging conditions a system needs to function, its recorded ways of failing. That clinicians should not be made into engineers is true and beside the point; the fitting comparison is pharmacological. A prescriber owes no account of how a drug is synthesised, yet is held to know its indications, its contraindications and its harms. A diagnostic algorithm set loose on patients warrants no less, and is at present the one clinical instrument exempted from that expectation.

## 2 Conclusion

Algorithmic diagnosis has done more than place a sharper instrument in the clinician's hand. It has altered the terms on which a clinician may hold any epistemic position at all.

A colleague who ventures an opinion can be questioned, contradicted, held to account. A deployed model undertakes nothing, betrays nothing, and errs by whole classes of patients at a stroke; its certification holds of a population where the clinician's decision holds of a person. Trust in the apparatus that produced it may be wholly warranted and settle nothing about the patient in the room. A curriculum cultivating calibrated trust in the output has been teaching the surrender of the validation it set out to secure.

Reconstructed on these lines, metacognition withstands the objection that has told most heavily against it. The evidence bears on the bare disposition to reflect and leaves untouched the knowledge a clinician holds of the conditions under which their own cognition, and now their instrument's, may be relied upon. Such knowledge can be taught and can be examined. Whether it can be exercised lies beyond the curriculum's reach, for interrogation needs an instrument and the minutes to use it, and a supervision that cannot be exercised is a supervision in name.

Medicine has always sought clinicians who can think independently when they must. That aim now requires teaching them to think about their patients and about the epistemic conditions under which they are thinking, including the conditions imposed by the systems sharing their diagnostic workspace. Such thinking matters most where those systems perform worst: the atypical patient, the edge of the distribution, the failure the manufacturer did not foresee. It is for those patients above all that a clinician must still be able to say what they know, and how they know it.

### 2.1 Positioning

The first author is a medical retina specialist whose clinical work is substantially image‐based, with training in the history of medicine and a research interest in the ethical and epistemological dimensions of clinical practice. The senior author is a researcher in metacognition and higher‐order thinking.

## Author Contributions

**Joel Hanhart:** conceptualization, methodology, writing – original draft, writing – review and editing. **Anat Zohar:** conceptualization, supervision, writing – review and editing. Both authors have read and approved the final manuscript.

## Funding

The authors have nothing to report.

## Ethics Statement

The authors have nothing to report.

## Conflicts of Interest

The authors declare no conflicts of interest.

## Artificial Intelligence Use

The authors used AI‐assisted tools for manuscript drafting and revision. All content was critically reviewed, edited and approved by the authors, who retain full responsibility for the final text.

## Data Availability

The authors have nothing to report.
